# Moderators of the association between regular smoking exposure and motivation and attempts to quit: a repeat cross‐sectional study

**DOI:** 10.1111/add.15479

**Published:** 2021-03-31

**Authors:** Harry Tattan‐Birch, Jamie Brown, Loren Kock, Lion Shahab

**Affiliations:** ^1^ Department of Behavioural Science and Health University College London 1‐19 Torrington Place London UK

**Keywords:** Inequalities, motivation, nicotine dependence, peer influence, quit attempts, smoking exposure, socio‐economic position, tobacco control

## Abstract

**Aims:**

To estimate the associations between regular exposure to smoking by other people and motivation and attempts to quit among current smokers. To examine whether socio‐demographic and other factors moderate these associations.

**Design:**

A repeat nationally representative cross‐sectional survey. Data were collected monthly between November 2014 and February 2019.

**Setting:**

England.

**Participants:**

Current smokers ≥16 years of age from the Smoking Toolkit Study (*n* = 15 136).

**Measurements:**

Participants were asked whether other people regularly smoke in their presence, how motivated they were to quit and whether they had made a quit attempt in the past year. Moderators assessed were occupation‐based social grade, housing tenure, urges to smoke, high‐risk alcohol consumption, and disability. Adjusted analyses included moderators, socio‐demographic (age/sex/ethnicity/sexual orientation/marital status/children in household) and seasonal (quarter/year) confounders.

**Findings:**

Current smokers who were regularly exposed to other people smoking in their presence were less likely to be highly motivated to quit (OR = 0.88 [95% CI 0.80–0.97]), but were no less likely to have made a quit attempt in the past year (OR 1.04 [0.97–1.13], Bayes Factor (BF) = 0.05). The inverse relationship between regular smoking exposure and motivation to quit was moderated by urges to smoke, such that exposure was only associated with a reduction in motivation among those without strong urges to smoke (OR 0.83 [0.75–0.93] versus OR 1.04 [0.86–1.26]; *P* = 0.048). None of the other factors significantly moderated the association with motivation to quit, and none moderated the relationship between regular smoking exposure and quit attempts. All non‐significant interactions, except social grade (BF = 1.44) with quit attempts, had Bayes Factors that supported the hypothesis of no moderation (BF range: 0.12–0.21).

**Conclusions:**

Among current smokers in England, regular exposure to other smokers appears to be associated with lower motivation to quit in people without strong urges to smoke, yet there appears to be no association with quit attempts in the previous year. Social grade, housing tenure, high‐risk alcohol consumption and disability do not moderate these associations.

## Introduction

People who are regularly exposed to smoking by others are more likely to start smoking themselves [1, 2]. After starting, they have a lower interest in quitting and are less likely to successfully quit [3, 4]. This ‘regular smoking exposure’ increases contact with cues that can induce urges to smoke, such as the sight and smell of cigarettes, which may undermine quit attempts [5, 6]. It might also increase social pressure to smoke, both via direct persuasion and through the influence of social norms [7]. However, this relationship between regular smoking exposure and quitting may depend on several risk factors. Using data from the Smoking Toolkit Study (STS), we investigated the associations that smoking exposure had with motivation and attempts to quit. We also examined whether socio‐demographic and behavioural factors moderated these associations.

Although the overall rate of smoking in England has declined over the past few decades, there remains a higher prevalence among disadvantaged (25.9%) compared with more affluent socioeconomic groups (10.2%) [8]. Moreover, smoking rates are higher in people with disabilities [9]. This gradient in smoking prevalence means that the burden of smoking‐related mortality and morbidity is also greatest in disadvantaged groups [10, 11, 12] and, as such, smoking remains a leading cause of health inequalities.

The association between regular smoking exposure and motivation to quit may be exacerbated by disadvantage. For example, in interviews with smokers, only those from working class occupations experienced significant social pressure to smoke [13]. If the association between regular smoking exposure and motivation and attempts to quit depends on this social pressure, it may be moderated by occupation‐based social grade [14]. Smoking exposure may also have a greater effect on people with disabilities, some of whom would be less able to remove themselves from environments that propagate tobacco use [15, 16].

In addition, adults in social housing have twice the odds of being a smoker when compared to those in other forms of housing [17]. In areas with high smoking rates, it can be considered the norm to smoke—that further amplifies its prevalence [18, 19]. Therefore, groups in social housing likely have stronger smoking social norms, which could lead to a greater negative association between regular smoking exposures and motivation and attempts to quit.

Cigarette dependence —operationalised here as strength of urges to smoke—is another factor that may moderate the relationship between smoking exposure and motivation and attempts to quit. When shown smoking cues in experimental settings, individuals with higher cigarette dependence experience stronger urges to smoke [20]. Proximity to other people smoking may act as a cue for smokers, which elicits greater urges to smoke in those who are highly dependent. Alternatively, there could be a ceiling effect, whereby highly dependent individuals are likely to continue smoking regardless of their exposure to other people smoking. Conversely, those who are less dependent and usually have weaker urges to smoke may be more influenced by exposure to smoking in their social environment.

Consuming alcohol also influences smoking cue‐reactivity. In experiments, drinking alcohol increases participants’ urges to smoke after being exposed to smoking cues [21]. Moreover, individuals who have high alcohol consumption may be particularly susceptible to peer influence [22]. Therefore, those with high‐risk alcohol consumption might be especially influenced by being near other people smoking.

It is important to understand which groups may be most influenced by smoking exposure, as this could help targeting of interventions and regulation. For instance, if people who live in social housing are especially influenced by smoking exposure, this would indicate that implementing smokefree policies and targeted cessation support in these areas may help to promote quitting and improve motivation to quit. Similarly, if smoking exposure has a disproportionally stronger association with quit attempts and motivation to quit among disadvantaged individuals, broader smokefree policies may be an effective intervention to help reduce smoking‐related health inequalities.

This study aims to investigate whether the association of regular smoking exposure with motivation to quit smoking and incidence of a quit attempt is moderated by these key socio‐demographic and behavioural factors. Specifically, we aim to answer the following research questions:

1. Among current smokers, what is the association between regular smoking exposure and motivation to quit, before and after adjusting for potential confounders?
2. Among current smokers, what is the association between regular smoking exposure and the incidence of a quit attempt in the last 12 months, before and after adjusting for potential confounders?
3. Are these associations moderated by occupation‐based social grade, housing tenure, strength of urges to smoke, high‐risk alcohol consumption or disability, before and after adjusting for potential confounders?

## Methods

### Design and setting

Data were taken from the STS; a monthly cross‐sectional survey of the population in England age 16+. It used a hybrid of random probability and quota sampling. Grouped output areas—each containing 300 households—that were stratified by region and socio‐demographic characteristics were randomly selected for an interviewer visit. Interviewers chose houses within these areas that were most likely to fulfil their quotas, conducting face‐to‐face interviews with one member per house. The STS recruits participants that have a similar socio‐demographic composition to those of large‐scale national surveys with probability sampling (Health Survey of England) [23]. STS data were used between November 2014 (earliest date when smoking exposure was measured) and February 2019.

### Ethical approval

Ethical approval for the STS was granted originally by the University College London (UCL) Ethics Committee (ID 0498/001). The data were not collected by UCL and were anonymised before being received by UCL.

### Outcomes

Past year quit attempt was measured with the following question: ‘How many serious attempts to stop smoking have you made in the last 12 months? By serious attempt I mean you decided that you would try to make sure you never smoked again. Please include any attempt that you are currently making and please include any successful attempt made within the last year’.

Those who reported none were coded 0. Those who reported one or more were coded 1.

Motivation to quit was measured using the Motivation to Stop Scale (MTSS), which has been verified as an externally valid predictor of quit attempts in the next 12 months [24]. Individuals who reported that they intended to quit in the next 3 months were classified as having a high motivation to quit, as people who report this are substantially more likely to subsequently make a quit attempt [25].

### Explanatory variables

Regular smoking exposure participants were asked; ‘Other than yourself, does anyone regularly smoke cigarettes or use an e‐cigarette in your presence, such as at your home, work, car or other places that you visit regularly?’ Responses that reported regular exposure to cigarette smoking were coded 1, whereas those with no exposure or only exposure to e‐cigarettes were coded 0. The question also asked about e‐cigarette exposure to ensure that participants did not mistakenly equate electronic with conventional cigarettes.

### Moderators

Social grade, operationalised as a variable for socio‐economic position, was measured using the National Readership Survey classification [26]. This tool measures the occupation of the chief income earner. Occupational groups were dichotomised into ABC1 (managerial, administrative and professional occupations; coded 0) versus C2DE (manual and casual workers, state pensioners and unemployed; coded 1).

Housing tenure, this variable was also split into two groups; those living in social housing (coded 1) and those in other accommodation (coded 0).

Strength of urges to smoke was measured using the strength of urges score, in which participants were asked, ‘How strong have the urges to smoke been in the past 24 hours? Slight, moderate, strong, very strong, extremely strong’. Those who reported having strong, very strong or extremely strong urges were classified as having strong urges to smoke (coded 1). All others were coded zero. This measure is used here as a proxy for cigarette dependence. Previous research from the Smoking Toolkit Study found that strength of urges is a better predictor of relapse after a quit attempt than other measures of cigarette dependence [23].

High‐risk alcohol consumption was identified as those who scored greater than or equal to five on the three consumption questions of the Alcohol Use Disorders Identification Test (AUDIT‐C), which measures the typical frequency and amount of alcohol consumption, and frequency of binge drinking [25].

Disability was self‐reported disability and was measured with the question, ‘Do you consider yourself to have a disability within the meaning of the Disability Discrimination Act 1995?’ (yes/no).

### Covariates

The following potential confounders were selected a priori: female (yes/no); age (16–24, 25–34, 35–44, 45–54, 55–64, 65+); ethnicity (White/not White); sexual orientation (heterosexual/not heterosexual); marital status (married/not married); children in household (yes/no); quarter of survey; and year of survey. These covariates have known or plausible associations with explanatory variables and outcomes [27, 28].

The variables that are listed as moderators were also entered as covariates in the logistic regression models testing all other moderators.

### Analyses

The analysis included complete cases who reported that they regularly or occasionally smoke cigarettes (including roll‐your‐own) or another tobacco product. Data were analysed in R [29]. The pre‐analysis plan and analysis code is available on the Open Science Framework (OSF) (https://osf.io/w5j8z/).

We reported the number and percentage of participants who gave each response to questions listed in the measures section. We also reported descriptive statistics for complete cases (i.e. participants who responded to every question).

The associations between regular smoking exposure and the two outcomes—incidence of a quit attempt and high motivation to quit—were assessed in separate univariable logistic regression models. To examine the independent association after adjustment for potential confounders, multivariable logistic regression models for each outcome were constructed also including all moderators and quarter and year of survey. To examine moderation effects, a series of models were constructed including each socio‐demographic factor, smoking exposure and their two‐way interaction. This series of models were produced for both outcomes, and also repeated with the inclusion of all other moderators and covariates.

We reported ORs and 95% CIs for these analyses and, for non‐significant results, Bayes factors were calculated. Following a conservative approach, we modelled the alternate hypotheses as half‐normal distributions centred on zero, with a standard deviation equal to the expected effect size [30]. Based on previous research into barriers to quit attempts, the expected effect size was set as the natural logarithm of OR = 0.70 [31, 32]. A robustness region was calculated for each Bayes factor, which specified the range of expected effect sizes used when constructing the alternative hypothesis that support the same conclusion (i.e. support for the null hypothesis) [33].

Two sets of sensitivity analyses were conducted. First, analyses for motivation to quit were repeated using multiple linear regressions with continuous MTSS score as the outcome. Second, analyses for quit attempts were repeated using gamma‐Poisson regression with counts of the number of past‐year quit attempts as the outcome. A pre‐registered sensitivity analysis using a continuous measure of quit attempts was corrected following comments from the statistical editor and removed from this article, but it is available on the OSF (https://osf.io/w5j8z/).

## Results

Of the 15 790 current smokers who were surveyed, 15 136 (95.9%) provided a complete set of responses. Characteristics for the full sample with pairwise deletion of data and the complete cases sample are shown in Table 1.

**Table 1:** Sample characteristics.

	*All adults (n = 15 790), n (%)*	*Complete cases (n = 15 136), n (%)*
**Smoking characteristics**		
Quit attempt		
None	11 542 (73.1)	11 068 (73.1)
One or more	4248 (26.9)	4068 (26.9)
Motivation to quit		
Low	13 474 (85.3)	12 910 (85.3)
High	2316 (14.7)	2226 (14.7)
Smoking exposure		
Not exposed	5264 (33.3)	5020 (33.2)
Exposed	10 526 (66.7)	10 116 (66.8)
**Socio‐demographic characteristics**		
Socioeconomic position		
ABC1	6405 (40.6)	6149 (40.6)
C2DE	9385 (59.4)	8987 (59.4)
Housing tenure		
Other	10 836 (69.1)	10 520 (69.5)
Social	4841 (30.9)	4616 (30.5)
Urges to smoke		
Weak	11 876 (75.5)	11 435 (75.5)
Strong	3850 (24.5)	3701 (24.5)
High risk alcohol consumption		
No	10 108 (64.6)	9759 (64.5)
Yes	5540 (35.4)	5377 (35.5)
Disability		
No	12 869 (83.1)	12 594 (83.2)
Yes	2615 (16.9)	2542 (16.8)
Sex		
Woman	7408 (46.9)	7112 (47.0)
Not woman	8382 (53.1)	8024 (53.0)
Age		
16–24	2935 (18.6)	2797 (18.5)
25–34	3134 (19.8)	3015 (19.9)
35–44	2521 (16.0)	2447 (16.2)
45–54	2668 (16.7)	2561 (16.9)
55–64	2315 (14.7)	2197 (14.5)
65+	2217 (14.0)	2119 (14.0)
Ethnicity		
Not White	1669 (10.6)	1576 (10.4)
White	14 045 (89.4)	13 560 (89.6)
Sexual orientation		
Not heterosexual	1484 (9.6)	1358 (9.0)
Heterosexual	14 209 (90.5)	13 778 (91.0)
Marital status		
Not married	8678 (55.0)	8239 (54.4)
Married	7112 (45.0)	6897 (45.6)
Children in household		
No	10 773 (68.2)	10 273 (67.9)
Yes	5017 (31.8)	4863 (32.1)
**Survey characteristics**		
Quarter of survey		
Q1	4112 (26.0)	3955 (26.1)
Q2	3805 (24.1)	3623 (23.9)
Q3	3625 (23.0)	3486 (23.0)
Q4	4248 (26.9)	4072 (26.9)
Year of survey		
2014	670 (4.2)	630 (4.2)
2015	3914 (24.8)	3782 (25.0)
2016	3706 (23.5)	3535 (23.4)
2017	3410 (21.6)	3270 (21.6)
2018	3550 (22.5)	3393 (22.4)
2019	540 (3.4)	526 (3.5)

The majority (66.8%) of participants reported regular exposure to others smoking in their presence. In unadjusted analyses, those who were regularly exposed to smoking were less likely to have a high motivation to quit than those who were not (OR 0.88, 95% CI 0.80–0.97, *P* < 0.01, Table 2). However, the odds of making a quit attempt in the last 12 months did not differ between these groups (OR 1.04, 95% CI 0.97–1.13, *P* = 0.27, BF 0.05). Table 2 shows results were adjusted for moderators, year, and quarter of survey, alongside interactions between smoking exposure and moderators.

**Table 2:** Associations between regular smoking exposure and motivation and attempts to quit.

	***Motivation to quit***						***Quit attempts***					
	*OR*_*un*_ ^*a*^ *[95% CI]*	*P*	*Bayes Factor*	*OR*_*adj*_ ^*a*^ *[95% CI]*	*P*	*Bayes factor*	*OR*_*un*_ *[95% CI]*	*P*	*Bayes factor*	*OR*_*adj*_ *[95%CI]*	*P*	*Bayes factor*
Smoking exposure												
No	1.00	–	–	1.00	–	–	1.00	–	–	1.00	–	–
Yes	0.88 [0.80–0.97]	0.009	–	0.85 [0.78–0.94]	.002	–	1.04 [0.97–1.13]	0.27	0.05	0.98 [0.91–1.07]	0.72	0.15
Social grade												
ABC1	1.00	–	–	1.00	–	–	1.00	–	–	1.00	–	–
C2DE	0.77 [0.67–0.90]	<0.001	–	0.72 [0.62–0.85]	<0.001	–	0.97 [0.86–1.10]	0.65	–	0.87 [0.76–0.99]	0.04	–
Interaction with exposure	1.00 [0.83–1.21]	0.995	0.26	1.00 [0.83–1.21]	0.998	0.26	0.87 [0.75–1.02]	0.08	1.78	0.88 [0.75–1.03]	0.11	1.44
Housing tenure												
Other	1.00	–	–	1.00	–	–	1.00	–	–	1.00	–	–
Social	0.97 [0.82–1.14]	0.68	–	0.96 [0.81–1.14]	0.64	–	1.11 [0.97–1.27]	0.12	–	1.07 [0.93–1.24]	0.32	–
Interaction with exposure	1.06 [0.87–1.31]	0.55	0.19	0.96 [0.88–1.34]	0.42	0.17	1.05 [0.89–1.24]	0.56	0.15	1.08 [0.92–1.28]	0.36	0.13
Urges to smoke												
Weak	1.00	–	–	1.00	–	–	1.00	–	–	1.00	–	–
Strong	0.95 [0.79–1.13]	0.54	–	0.96 [0.80–1.15]	0.67	–	1.24 [1.07–1.43]	0.004	–	1.25 [1.08–1.44]	0.003	‐
Interaction with exposure	1.25 [1.00–1.56]	0.048	–	1.27 [1.02–1.58]	0.04	–	1.09 [0.91–1.30]	0.34	0.13	1.11 [0.93–1.32]	0.27	0.12
Alcohol consumption												
Low risk	1.00	–	–	1.00	–	–	1.00	–	–	1.00	–	–
High risk	0.93 [0.79–1.10]	0.43	‐	0.91 [0.76–1.07]	0.26	‐	0.92 [0.80–1.06]	0.25	‐	0.90 [0.78–1.04]	0.15	0.98
Interaction with exposure	1.00 [0.82–1.23]	0.99	0.27	1.04 [0.85–1.28]	0.71	0.21	1.00 [0.85–1.18]	0.99	0.23	1.02 [0.86–1.21]	0.82	0.20
Disability												
No	1.00	–	–	1.00	–	–	1.00	–	–	1.00	–	–
Yes	0.93 [0.76–1.13]	0.48	–	1.04 [0.84–1.28]	0.70	–	1.08 [0.92–1.27]	0.35	–	1.20 [1.01–1.42]	0.03	–
Interaction with exposure	1.21 [0.94–1.55]	0.14	0.14	1.19 [0.92–1.53]	0.18	0.15	1.16 [0.95–1.41]	0.15	0.12	1.14 [0.93–1.40]	0.20	0.13

^a^ OR_un_ are unadjusted odds ratios. OR_adj_ are odds ratios that have been adjusted for gender, age, ethnicity, sexual orientation, marital status, children in household, quarter of survey, and year of survey, alongside all the potential moderators listed in this table.

**Figure 1:**
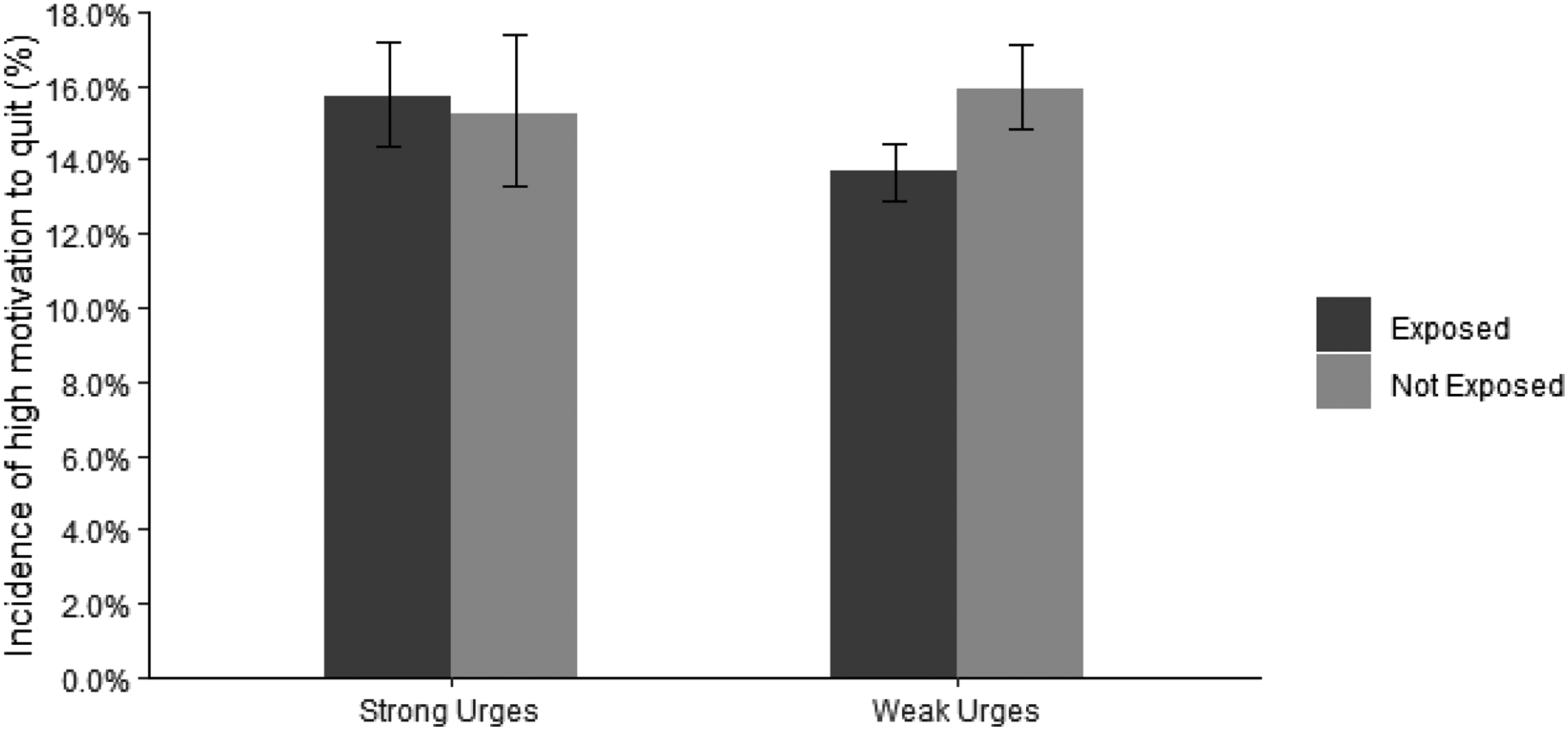
Incidence of a high motivation to quit by strength of urges to smoke and regular smoking exposure. Estimates are unadjusted for covariates and error bars represent 95% CI

Strength of urges to smoke moderated the association between smoking exposure and motivation to quit such that exposure only reduced the incidence of high motivation to quit among those who experience strong urges to smoke, as shown in Fig. 1 (OR_un_ 0.83, 95% CI 0.75–0.93 vs. OR_un_ 1.04, 95% CI 0.86–1.26).

None of the other socio‐demographic or behavioural factors significantly moderated the association between regular smoking exposure and motivation to quit in either adjusted or unadjusted models (Table 2). Moreover, none of these factors significantly moderated the relationship between exposure and quit attempts. Bayes factors indicated there was insufficient evidence on whether the association between regular smoking exposure and quit attempts was moderated by social grade (BF 1.44). However, there was moderate evidence for the null hypothesis (no moderation) for all other socio‐demographic factors, irrespective of the outcome (BF range 0.12–0.26). When calculating these Bayes factors, a null of OR 1.00 was compared to an expected effect size of OR 0.7. Planned sensitivity analyses showed that our conclusions were robust to changes in this assumption—the expected effect size could be altered to OR 0.76–0.89 while still showing support for the null hypothesis. However, we cannot rule out the possibility of small moderation effects.

Further sensitivity analyses (Table S1 and Table S2) explored whether similar results would be found when the outcomes were continuous MTSS score for motivation to quit and counts for quit attempts. In both unadjusted and adjusted analyses, there were no significant relationships between regular smoking exposure, MTSS score and number of quit attempts. We also found that—in both adjusted and unadjusted analyses—none of the socio‐demographic factors significantly interacted with exposure when predicting quit attempts or motivation to quit. However, in the analyses that used a continuous measure of MTSS score, the assumption of normality of residuals was violated.

## Discussion

Current smokers who were regularly exposed to smoking by other people were equally likely to have made a quit attempt in the past year but were less likely to have a high current motivation to quit than those who were not. This negative association with motivation was only evident in those without strong urges to smoke. Social grade, housing tenure, high‐risk alcohol consumption and disability did not moderate the relationship that smoking exposure has with motivation and attempts to quit.

Our results suggest that there may be a negative effect of regular smoking exposure on motivation to quit. This is consistent with previous research showing that smokers who have close friends that smoke have a lower intention to quit [34]. A possible mechanism is that smokers who are regularly exposed to other people smoking encounter social pressure to smoke and a greater number of smoking cues [3, 5, 7].

Our results suggest no effect of regular smoking exposure on quit attempts. One possible explanation is the focus of this paper on current smokers, which could have introduced collider bias [35]. Previous studies have shown [34] that having close friends who smoke reduces the likelihood that an individual will quit successfully. Among those who made a quit attempt, this would have caused more non‐exposed individuals to be removed from the analysis, leading us to underestimate the association between regular smoking exposure and quit attempts. Further research that includes responses from ex‐smokers or explores longitudinal associations is needed to determine the plausibility of this interpretation.

Strength of urges to smoke moderated the association between regular smoking exposure and motivation to quit, such that the association was only evident among participants without strong urges to smoke. A possible explanation for this is that more dependent smokers who experience strong urges to smoke may be less influenced by environmental and social forces and, therefore, smoking exposure has little influence on their motivation to quit. In the sensitivity analysis that used a continuous measure of motivation to quit, the interaction between smoking exposure and strength of urges was non‐significant. However, the assumption of normality of residuals was violated, so results from the primary analysis are likely more robust. Nonetheless, this reduces the certainty of this finding.

To our knowledge, this study is the first to explore how the association between regular smoking exposure with motivation and attempts to quit may differ for those in advantaged and disadvantaged socio‐demographic groups. We were able show the striking similarity in the association of regular smoking exposure with motivation and attempts to quit across social grade and housing tenure—a finding not previously reported in the literature. This suggests that higher smoking prevalence in these disadvantaged groups may not be the result of a differential impact of smoking exposure on quitting. However, as we only measured motivation and attempts to quit, it is possible that there is a greater negative association between smoking exposure and quit success among disadvantaged groups. This would mirror previous findings that socio‐demographic factors are associated with quit success but not attempts [36]. Nonetheless, targeted regulation (e.g. smokefree zones) may still have an equity positive impact on smoking‐related inequalities, as smoking prevalence is much higher among disadvantaged groups.

This study had a number of strengths. The sample was representative of smokers in England and large enough to detect small associations, which allowed us to disambiguate null findings. Moreover, we were able to control for putative confounding variables. Several limitations arise from the cross‐sectional design, such as our inability to (i) provide evidence for a temporal association between outcomes and predictors and (ii) rule out self‐selection bias. Because of this, it is possible smokers who were less motivated to quit could have been more likely to expose themselves to smoking peers. Another limitation is that only current smokers were included in the analysis, which meant that we could not investigate the relationship between smoking exposure and quit success. Finally, smoking exposure was measured on a dichotomous scale, with participants reporting either being regularly exposed or not. This meant we could not investigate the source or severity of exposure. It is possible that exposure to smoking from peers has a greater impact on smoking behaviours than exposure from other people [37]. Moreover, there may only be a negative association (and/or moderation by socioeconomic position) between smoking exposure and quitting behaviours among those with more persistent exposure. Further research should investigate different sources and severities of smoking exposure to identify how these may influence quitting trajectories, alongside how their effects vary across socio‐demographic groups.

## Conclusions

Among current smokers in England, regular exposure to other smokers was associated with lower motivation to quit in people without strong urges to smoke, yet there was no association with quit attempts in the previous year. The lack of moderation by other included covariates suggests that the association between regular smoking exposure and motivation and attempts to quit is similar across people with various behavioural and socio‐demographic characteristics.

## Declaration of interests

Authors declare no financial links with tobacco companies, e‐cigarette manufacturers or their representatives. L.S. has received honoraria for talks, an unrestricted research grant and travel expenses to attend meetings and workshops by pharmaceutical companies that make smoking cessation products (Pfizer, Johnson and Johnson). J.B. has received unrestricted research funding from Pfizer.

## Author contributions

**Harry Tattan‐Birch:** Conceptualization‐Equal, Formal analysis‐Lead, Methodology‐Equal, Writing‐original draft‐Lead, Writing‐review & editing‐Equal. **Jamie Brown:** Conceptualization; data curation; funding acquisition; methodology; supervision. **Loren Kock:** Conceptualization; supervision. **Lion Shahab:** Conceptualization; funding acquisition; methodology; supervision.

## Supporting information


**Table S1** Sensitivity analysis using continuous measure of motivation to quit.
**Table S2** Sensitivity analysis modelling number of quit attempts in a gamma‐Poisson (negative‐binomial) regression.

## References

[1] Flay B. R. , Hu F. B. , Siddiqui O. , Day L. E. , Hedeker D. , Petraitis J. , *et al*. Differential influence of parental smoking and friends' smoking on adolescent initiation and escalation of smoking. J Health Soc Behav 1994; 35: 248–265. 7983337

[2] Okoli C. T. C. , Kodet J. A systematic review of secondhand tobacco smoke exposure and smoking behaviors: smoking status, susceptibility, initiation, dependence, and cessation. Addict Behav Elsevier Ltd 2015; 47: 22–32. 25863004 10.1016/j.addbeh.2015.03.018

[3] Yong L. C. , Luckhaupt S. E. , Li J. , Calvert G. M. Quit interest, quit attempt and recent cigarette smoking cessation in the US working population, 2010. Occup Environ Med [Internet] 2014 [cited 2019 Mar 13]; 71: 405–414. Available at: http://www.ncbi.nlm.nih.gov/pubmed/24497440 24497440 10.1136/oemed-2013-101852 PMC4528304

[4] Eng L. , Su J. , Qiu X. , Palepu P. R. , Hon H. , Fadhel E. , *et al*. Second‐hand smoke as a predictor of smoking cessation among lung cancer survivors. J Clin Oncol [Internet] 2014 [cited 2021 Feb 25]; 32: 564–570. Available at: http://ascopubs.org/doi/10.1200/JCO.2013.50.9695 24419133 10.1200/JCO.2013.50.9695

[5] Shiffman S. , Dunbar M. , Kirchner T. , Li X. , Tindle H. , Anderson S. , *et al*. Smoker reactivity to cues: effects on craving and on smoking behavior. J Abnorm Psychol [Internet] 2013 [cited 2019 Mar 13]; 122: 264–280. Available at: http://www.ncbi.nlm.nih.gov/pubmed/22708884 22708884 10.1037/a0028339 PMC3988583

[6] Carter B. L. , Tiffany S. T. Meta‐analysis of cue‐reactivity in addiction research. Addiction [Internet] 1999 [cited 2019 Mar 13]; 94: 327–340. Available at: http://doi.wiley.com/10.1046/j.1360-0443.1999.9433273.x 10605857

[7] van den Putte B. , Yzer M. C. , Brunsting S. Social influences on smoking cessation: a comparison of the effect of six social influence variables. Prev Med (Baltim) [Internet] 2005 [cited 2019 Mar 13]; 41: 186–193. Available at: https://linkinghub.elsevier.com/retrieve/pii/S0091743504005651 10.1016/j.ypmed.2004.09.040 15917010

[8] Office for National Statistics . Adult smoking habits in the UK: 2017. ONS Statistical Bulletin 2018.

[9] Emerson E. Smoking among adults with and without disabilities in the UK. J Public Health (Bangkok) [Internet] 2018 [cited 2019 Mar 18]; 40: e502–e509. Available at: https://academic.oup.com/jpubhealth/article/40/4/e502/4958209 10.1093/pubmed/fdy062 29617853

[10] Jha P. , Peto R. , Zatonski W. , Boreham J. , Jarvis M. J. , Lopez A. D. Social inequalities in male mortality, and in male mortality from smoking: indirect estimation from national death rates in England and Wales, Poland, and North America. Lancet [Internet] 2006 [cited 2019 Mar 13]; 368: 367–370. Available at: http://www.ncbi.nlm.nih.gov/pubmed/16876664 16876664 10.1016/S0140-6736(06)68975-7

[11] Hiscock R. , Dobbie F. , Bauld L. Smoking cessation and socioeconomic status: an update of existing evidence from a National Evaluation of English stop smoking services. Biomed Res Int [Internet] 2015 [cited 2019 Feb 26]; 2015: 274056. Available at: http://www.ncbi.nlm.nih.gov/pubmed/26273602 26273602 10.1155/2015/274056 PMC4529910

[12] Hovanec J. , Siemiatycki J. , Conway D. I. , Olsson A. , Stücker I. , Guida F. , *et al*. Lung cancer and socioeconomic status in a pooled analysis of case‐control studies. Van Wouwe JP, editor. PLoS One [Internet] 2018 [cited 2019 Mar 13]; 13: e0192999. Available at: http://www.ncbi.nlm.nih.gov/pubmed/29462211 29462211 10.1371/journal.pone.0192999 PMC5819792

[13] Chamberlain K. , O'neill D. Understanding social class differences in health: a qualitative analysis of smokers' health beliefs. Psychol Health [Internet] 1998 [cited 2019 Mar 13]; 13: 1105–1119. Available at: http://www.tandfonline.com/doi/abs/10.1080/08870449808407453

[14] Green H. D. , Horta M. , de la Haye K. , Tucker J. S. , Kennedy D. R. , Pollard M. , *et al*. Peer influence and selection processes in adolescent smoking behavior: a comparative study. Nicotine Tob Res [Internet] 2013 [cited 2019 Mar 13]; 15: 534–541. Available at: http://www.ncbi.nlm.nih.gov/pubmed/22944605 22944605 10.1093/ntr/nts191 PMC3612003

[15] Wang P. P. , Badley E. M. , Gignac M. Exploring the role of contextual factors in disability models. Disabil Rehabil [Internet] 2006 [cited 2019 Mar 13]; 28: 135–140. Available at: http://www.tandfonline.com/doi/full/10.1080/09638280500167761 16393844 10.1080/09638280500167761

[16] Enabling America [Internet]. Washington, D.C.: National Academies Press; 1997 [cited 2019 Mar 13]. Available at: http://www.nap.edu/catalog/5799

[17] Jackson S. E. , Smith C. , Cheeseman H. , West R. , Brown J. Finding smoking hot‐spots: a cross‐sectional survey of smoking patterns by housing tenure in England. Addiction [Internet] 2019 [cited 2019 Apr 5]; 114: 889–895. Available at: http://doi.wiley.com/10.1111/add.14544 30597650 10.1111/add.14544 PMC6491989

[18] Shaw M. Housing and public health. Annu Rev Public Health [Internet] 2004 [cited 2019 Apr 5]; 25: 397–418. Available at: http://www.annualreviews.org/doi/10.1146/annurev.publhealth.25.101802.123036 15015927 10.1146/annurev.publhealth.25.101802.123036

[19] Jackson S. E. , Proudfoot H. , Brown J. , East K. , Hitchman S. C. , Shahab L. Perceived non‐smoking norms and motivation to stop smoking, quit attempts, and cessation: a cross‐sectional study in England. Sci Rep 2020; 10: 10487. 32591555 10.1038/s41598-020-67003-8 PMC7320183

[20] Smolka M. N. , Bühler M. , Klein S. , Zimmermann U. , Mann K. , Heinz A. , *et al*. Severity of nicotine dependence modulates cue‐induced brain activity in regions involved in motor preparation and imagery. Psychopharmacology (Berl) [Internet] 2006 [cited 2019 Mar 13]; 184: 577–588. Available at: http://link.springer.com/10.1007/s00213-005-0080-x 16133128 10.1007/s00213-005-0080-x

[21] Burton S. M. , Tiffany S. T. The effect of alcohol consumption on craving to smoke. Addiction [Internet] 1997 [cited 2019 Mar 13]; 92: 15–26. Available at: http://www.ncbi.nlm.nih.gov/pubmed/9060194 9060194

[22] Trucco E. M. , Colder C. R. , Wieczorek W. F. Vulnerability to peer influence: a moderated mediation study of early adolescent alcohol use initiation. Addict Behav [Internet] 2011 [cited 2019 Mar 13]; 36: 729–736. Available at: https://www.sciencedirect.com/science/article/pii/S0306460311000797 21420241 10.1016/j.addbeh.2011.02.008 PMC3088763

[23] Fidler J. A. , Shahab L. , West O. , Jarvis M. J. , McEwen A. , Stapleton J. A. , *et al*. “The smoking toolkit study”: a national study of smoking and smoking cessation in England. BMC Public Health [Internet] 2011 [cited 2019 Mar 18]; 11: 479. Available at: http://bmcpublichealth.biomedcentral.com/articles/10.1186/1471-2458-11-479 21682915 10.1186/1471-2458-11-479 PMC3145589

[24] Hummel K. , Brown J. , Willemsen M. C. , West R. , Kotz D. External validation of the motivation to stop scale (MTSS): findings from the international tobacco control (ITC) Netherlands survey. Eur J Public Health [Internet] 2016 [cited 2019 Mar 13]; 27: ckw105. Available at: https://academic.oup.com/eurpub/article-lookup/doi/10.1093/eurpub/ckw105 10.1093/eurpub/ckw105 28177479

[25] Kotz D. , Brown J. , West R. Predictive validity of the motivation to stop scale (MTSS): a single‐item measure of motivation to stop smoking. Drug Alcohol Depend 2013; 128: 15–19. 22943961 10.1016/j.drugalcdep.2012.07.012

[26] Social Grade in National Readership Survey [Internet]. [cited 2019 Nov 7]. Available at: http://www.nrs.co.uk/nrs-print/lifestyle-and-classification-data/social-grade/

[27] Chandola T. , Head J. , Bartley M. Socio‐demographic predictors of quitting smoking: how important are household factors? Addiction [Internet] 2004 [cited 2020 Nov 26]; 99: 770–777. Available at: http://doi.wiley.com/10.1111/j.1360-0443.2004.00756.x 15139875 10.1111/j.1360-0443.2004.00756.x

[28] Beard E. , Brown J. , Jackson S. E. , West R. , Kock L. , Boniface S. , *et al*. Independent associations between different measures of socioeconomic position and smoking status: a cross‐sectional study of adults in England. Nicotine Tob Res [Internet] 2020 [cited 2020 Nov 26]; 2020: 1–8. Available at: https://academic.oup.com/ntr/advance-article/doi/10.1093/ntr/ntaa030/5728574 10.1093/ntr/ntaa030 PMC7789954 32026943

[29] R Development Core Team . R: A language and environment for statistical computing. 2008.

[30] Dienes Z. Using Bayes to get the most out of non‐significant results. Front Psychol [Internet] 2014 [cited 2019 Mar 13]; 5: 781. Available at: http://www.ncbi.nlm.nih.gov/pubmed/25120503 25120503 10.3389/fpsyg.2014.00781 PMC4114196

[31] Jackson S. E. , Beard E. , Michie S. , Shahab L. , Raupach T. , West R. , *et al*. Are smokers who are regularly exposed to e‐cigarette use by others more or less motivated to stop or to make a quit attempt? A cross‐sectional and longitudinal survey. BMC Med [Internet] 2018 [cited 2019 Apr 8]; 16: 206. Available at: https://bmcmedicine.biomedcentral.com/articles/10.1186/s12916-018-1195-3 30424771 10.1186/s12916-018-1195-3 PMC6234626

[32] Fidler J. A. , West R. Enjoyment of smoking and urges to smoke as predictors of attempts and success of attempts to stop smoking: a longitudinal study. Drug Alcohol Depend [Internet] 2011 [cited 2019 Apr 9]; 115: 30–34. Available at: https://www.sciencedirect.com/science/article/pii/S0376871610003613?via%3Dihub 21111539 10.1016/j.drugalcdep.2010.10.009

[33] Lakens D. , McLatchie N. , Isager P. M. , Scheel A. M. , Dienes Z. Improving inferences about null effects with Bayes factors and equivalence tests. J Gerontol Ser B [Internet] 2018 [cited 2019 Apr 9]. Available at: http://www.ncbi.nlm.nih.gov/pubmed/29878211 10.1093/geronb/gby065 29878211

[34] Hitchman S. C. , Fong G. T. , Zanna M. P. , Thrasher J. F. , Laux F. L. The relation between number of smoking friends, and quit intentions, attempts, and success: findings from the international tobacco control (ITC) four country survey. Psychol Addict Behav [Internet] 2014 [cited 2019 Feb 6]; 28: 1144–1152. Available at: http://www.ncbi.nlm.nih.gov/pubmed/24841185 24841185 10.1037/a0036483 PMC4266625

[35] Tattan‐Birch H. , Marsden J. , West R. , Gage S. H. Assessing and addressing collider bias in addiction research: the curious case of smoking and COVID‐19. Addiction [Internet] 2020 [cited 2020 Nov 27]. Available at: https://onlinelibrary.wiley.com/doi/10.1111/add.15348.add.15348 10.1111/add.15348 PMC7753816 33226690

[36] Kotz D. , West R. Explaining the social gradient in smoking cessation: It's not in the trying, but in the succeeding. Tob Control 2009 Feb 1; 18: 43–46. 18936053 10.1136/tc.2008.025981

[37] Wang M. Q. , Fitzhugh E. C. , Westerfield R. C. , Eddy J. M. Family and peer influences on smoking behavior among American adolescents: an age trend. J Adolesc Health [Internet] 1995 [cited 2019 May 3]; 16: 200–203. Available at: https://www.sciencedirect.com/science/article/pii/1054139X9400097X 7779829 10.1016/1054-139X(94)00097-X

