# Supplementary material for: Moderators of the association between regular smoking exposure and motivation and attempts to quit: a repeat cross‐sectional study
Source: Addiction. 2021 Mar 31;116(10):2837–46. doi: 10.1111/add.15479 (PMC8612132; doi:10.1111/add.15479)
Supplement: Supplementary file 1 — Table S1 Sensitivity analysis using continuous measure of motivation to quit. Table S2 Sensitivity analysis modelling number of quit attempts in a gamma‐Poisson (negative‐binomial) regression. [file ADD-116-2837-s001.pdf]

Table S.1: Sensitivity analysis using continuous measure of motivation to quit.

	B_{un} [95% CI]	p	B_{adj} [95% CI]	p
Smoking Exposure				
Main effect	-0.051 [-0.118 - 0.016]	.136	-0.005 [-0.072 - 0.063]	.217
Social Grade				
Main effect	0.168 [0.058 - 0.278]	.003	0.214 [0.102 - 0.325]	<.001
Int. with Exposure	0.077 [-0.058 - 0.212]	.265	0.073 [-0.061 - 0.207]	.284
Housing Tenure				
Main effect	-0.034 [-0.153 - 0.085]	.574	-0.040 [-0.161 - 0.081]	.522
Int. with Exposure	0.062 [-0.083 - 0.208]	.400	0.035 [-0.109 - 0.178]	.635
Urges to Smoke				
Main effect	-0.104 [-0.232 - 0.024]	.112	-0.117 [-0.244 - 0.010]	.070
Int. with Exposure	0.028 [-0.128 - 0.183]	.729	0.009 [-0.145 - 0.163]	.908
Alcohol Consumption				
Main effect	0.096 [-0.023 - 0.215]	.114	0.133 [0.013 - 0.252]	.029
Int. with Exposure	-0.075 [-0.218 - 0.068]	.305	-0.111 [-0.253 - 0.03]	.123
Disability				
Main effect	-0.015 [-0.158 - 0.127]	.834	-0.015 [-0.088 - 0.059]	.699
Int. with Exposure	0.042 [-0.134 - 0.219]	.640	0.058 [-0.117 - 0.232]	.517

Table S.2: Sensitivity analysis modelling number of quit attempts in a gamma-Poisson (negative-binomial) regression.

	RR_{un}* [95% CI]	p	RR_{adj}* [95%CI]	p
Smoking Exposure				
No	1.00	-	1.00	-
Yes	1.00 [0.93-1.07]	.952	0.96 [0.89-1.03]	.218
Social Grade				
ABC1	1.00	-	1.00	-
C2DE	1.04 [0.93-1.16]	.511	0.94 [0.84-1.06]	.325
Int. with Exposure	0.87 [0.76-1.01]	.060	0.88 [0.77-1.01]	.076
Housing Tenure				
Other	1.00	-	1.00	-
Social	1.09 [0.97-1.24]	.141	1.06 [0.93-1.20]	.393
Int. with Exposure	1.09 [0.94-1.26]	.270	1.09 [0.94-1.26]	.248
Urges to Smoke				
Weak	1.00	-	1.00	-
Strong	1.25 [1.10-1.42]	.001	0.94 [0.87-1.02]	.124
Int. with Exposure	1.08 [0.93-1.26]	.312	1.08 [0.92-1.26]	.347
Alcohol Consumption				
Low risk	1.00	-	1.00	-
High risk	0.89 [0.79-1.01]	.075	0.90 [0.79-1.01]	.083
Int. with Exposure	1.04 [0.90-1.21]	.602	1.04 [0.90-1.21]	.586
Disability				
No	1.00	-	1.00	-
Yes	1.20 [1.04-1.38]	.013	1.28 [1.11-1.47]	.001
Int. with Exposure	1.04 [0.88-1.24]	.634	1.03 [0.87-1.23]	.739

* RR_{un} are unadjusted rate ratios. RR_{adj} are rate ratios that have been adjusted for gender, age, ethnicity, sexual orientation, marital status, children in household, quarter of survey, and year of survey, alongside all the potential moderators listed in this table. Dispersion parameter θ ranged from 0.57 to 0.62 across models, indicating under-dispersion.
